# Physically aggressive behaviors in older people living with cognitive disorders: a systematic scoping review protocol

**DOI:** 10.1186/s13643-019-1091-8

**Published:** 2019-07-11

**Authors:** Anne Bourbonnais, Marie-Hélène Goulet, Philippe Landreville, Edith Ellefsen, Caroline Larue, Marie-Hélène Lalonde, Paul L. Gendreau

**Affiliations:** 1 Faculty of Nursing, Université de Montréal, Research Chair in Nursing Care for Older People and their Families, Research Centre of the Institut universitaire de gériatrie de Montréal, PO Box 6128, Station Centre-Ville, Montréal, Quebec H3C 3J7 Canada; 2 Faculty of Nursing, Université de Montréal, PO Box 6128, Station Centre-Ville, Montréal, Quebec H3C 3J7 Canada; 3 School of Psychology, Université Laval, 2325 Allée des Bibliothèques, Québec City, Quebec G1V 0A6 Canada; 4 Faculty of Medicine and Health Sciences, Université de Sherbrooke, 150 Place Charles-Le Moyne, Longueuil, Quebec J4K 0A8 Canada; 5 Faculty of Nursing, Université de Montréal, PO Box 6128, Station Centre-Ville, Montréal, Quebec H3C 3 J7 Canada; 6 Research Centre of the Institut universitaire de gériatrie de Montréal, 4565 Queen Mary, Montréal, Quebec H3W 1W5 Canada; 7 Department of Psychoeducation, Université de Montréal, PO Box 6128, Station Centre-Ville, Montréal, Quebec H3C 3J7 Canada

**Keywords:** Aggressive behaviors, Behavioral and psychological symptoms, Elderly, Neurocognitive disorders, Humanist perspective, Scoping review, Causes, Interventions, Consequences

## Abstract

**Background:**

Physically aggressive behaviors are very common among older people living with cognitive impairment. These behaviors may have significant consequences for family and formal caregivers, as well as for the other people in the older people’s environment, and are also a frequent cause of institutionalization. Two relevant systematic reviews have been published on the subject but do not specifically target physically aggressive behaviors or only focus on care in nursing homes. Moreover, they do not address the causes, associated factors, and consequences of these behaviors, even though these should indeed be considered when developing interventions. Thus, the purpose of this scoping review is to map the state of knowledge on these physically aggressive behaviors with a view to developing personalized interventions. Offering a humanist and relational perspective by which these behaviors may be examined, the Senses Framework will guide this review.

**Methods:**

The scoping review method of Levac, Colquhoun, and O’Brien will be used. Several databases (e.g., CINAHL, PubMed, PsycINFO, SCOPUS, Grey Literature Report, clinical trials registries) will be searched for literature published in the past 15 years, using a combination of keywords and descriptors. Other data sources will be used to identify non-indexed literature or unpublished results (e.g., articles references, journal tables of content, contact with key authors). The literature will be selected regardless of setting, if it concerns older people, aged 65, or older with cognitive impairment who present physically aggressive behaviors. Data will be extracted systematically by the research team. A quality assessment of the literature will be done to consider this aspect in the data synthesis. A content analysis will be used to synthesize the results.

**Discussion:**

No scoping review has been found on the physically aggressive behaviors of older people living with cognitive impairment in various settings. The results of this review will identify needs for further research and for clinical and training development on this problem from a humanist standpoint.

**Systematic review registration:**

Currently, it is not possible to register a systematic scoping review protocol (e.g., PROSPERO).

**Electronic supplementary material:**

The online version of this article (10.1186/s13643-019-1091-8) contains supplementary material, which is available to authorized users.

## Background

With between 11% and 68% of older people with cognitive impairment manifesting aggressive behaviors [1], this latter is indeed a common problem. These behaviors can be defined as overt expression, whether provoked or not, to hurt another living being [2]. Regardless of the environment, physically aggressive behaviors (PAB) have major consequences for family and formal caregivers, as well as for other people in the older person’s immediate surrounding, potentially resulting in physical and psychological harm. PAB are also a frequent cause of institutionalization for older people [2]. Personalized interventions are needed to decrease these consequences [3].

Two systematic reviews have been published to date that are relevant to PAB [4, 5]. The first addresses behaviors in general and does not examine PAB specifically [4]. The second covers the literature up until 2003 and focused on PAB in nursing homes [5]. To our knowledge, no review examining PAB in a home or hospital context has yet been published. Moreover, the causes, associated factors, and consequences of PAB are not addressed in the two reviews, even if these elements should be considered in the development of interventions.

To establish the current state of knowledge on PAB that could guide the development of personalized interventions, our systematic review will consider their causes, associated factors, and consequences, as well as existing interventions in the continuum from home care to nursing home care. To this end, our scoping review will be conducted systematically to map the state of knowledge of the PAB in older people with cognitive impairment. The examination, synthesis, and analysis of existing knowledge can be conducted extensively and comprehensively (e.g., many types of literature and many questions) in this type of review [6]. Rather than being limited to studies assessing the efficacy of interventions, this type of review includes all type of publications. Thus, it is possible to explore other elements for the development of personalized interventions. Four questions will guide this scoping review about the PAB of older people with cognitive impairment:

1. What factors are associated with their PAB?
2. What causes are described to explain their PAB?
3. What are the consequences of their PAB on the various stakeholders involved?
4. What interventions are considered effective to prevent and/or manage their PAB?

Developed to improve the care of older people, the Senses Framework [7] will guide this review. It stresses the importance of the mutual relationship between older people, family caregivers, and formal caregivers to provide human care. This framework also emphasizes that everyone’s needs must be considered to promote the well-being of each individual (for example, by fostering a sense of security). Guiding data extraction, it adds a dimension by which each publication will be examined: whether the relationship between the actors is considered and, if so, how. These data will strengthen our conclusions on the state of knowledge about which aspects should be considered in the development of personalized interventions.

## Methods

The scoping review method of Levac, Colquhoun, and O’Brien [8] will be used to map the state of knowledge on the PAB of older people with cognitive impairment. It includes all types of literature and has six steps: (1) identify the review questions, (2) identify the literature, (3) select the literature, (4) extract the data, (5) present the results, and (6) consult knowledge users. The scoping review will be conducted iteratively to adjust and refine the method throughout the study. This protocol will thus serve as a basis for highlighting and documenting the changes made. It is in keeping with the items proposed by the Preferred Reporting Items for Systematic Review and Meta-Analysis Protocols (PRISMA-P) [9] (see Additional file 1). Some of the items (1b, 2, 4, 13, 14, 15a, 15b, 15c, 16, 17) were not included, as they are not adapted to a scoping review. This protocol is not registered, since the International Prospective Register of Systematic Review (PROSPERO) does not yet allow for the registration of this type of systematic review.

### Eligibility criteria

To meet the aim of this scoping review, the literature that meets the following population-concept-context (PCC) criteria will be included in the review.

### Population

The analysis will include literature on individuals aged 65 and older with cognitive impairment. Cognitive impairment refers to major neurocognitive disorders as defined by the *Diagnostic and Statistical Manual of Mental Disorders* (DSM-5) [10] and can be caused by many types of degenerative disorders, including Alzheimer’s, frontotemporal lobar degeneration, Lewy body disease, and vascular disease.

### Concept

The core concept of this scoping review is PAB (defined above, in the “Background” section) exhibited by older people toward their peers, family caregivers, and/or formal caregivers. Literature on a group of behaviors will be included if it allows us to clearly distinguish results regarding PAB. Literature on self-harm, suicide attempts, aggressive behaviors in prison, and sexual aggression and assault by family or formal caregivers will be excluded. Literature about factors associated with PAB of older people with cognitive impairment, causes of these behaviors, and their consequences on the various stakeholders (e.g., family, formal caregivers) will be included. The literature on the effectiveness of interventions to prevent and/or manage PAB will also be included.

### Context

No restriction will be placed on where older people manifest PAB (e.g., at home, in a hospital, in a nursing home). Literature from all countries will be included.

### Type of records

The search strategy will be limited to literature, in English or French, published after 2003. This is because the only systematic review available on interventions for PAB was conducted on the literature published before this date. Also, many documents have been published on the PAB of older people with cognitive impairment since 2003. Authors often cited in the literature that will be selected for this scoping review, but published before 2003, and whose publications are still relevant will also be included. All types of literature will be considered. This includes, for example, primary studies (e.g., quasi-experimental, experimental, qualitative, and mixed-method designs), literature reviews (e.g., narrative reviews, meta-analysis, systematic reviews), gray literature (e.g., governmental reports, theses), and theoretical and opinion articles. Personal blogs and social media will be excluded.

### Information sources

As suggested by Cooper [11], four categories of sources will be targeted to identify the literature. First, many databases will be searched: Cumulative Index to Nursing and Allied Health Literature (CINAHL), PubMed, PsycINFO, Cochrane Clinical Trials, SCOPUS, Trip, ProQuest Dissertations, Epistemonikos, Grey Literature Report, clinical trials registries (e.g., ClinicalTrials.gov, Controlled-trials.com). The list of references will be examined, and a prospective search of key literature will be carried out in Web of Science. Second, the table of contents of the journals of the key articles will be examined. Third, governmental and organizational websites will be explored (e.g., Health Agencies, National Institute for Health and Care Excellence, Alzheimer’s Society, Alzheimer’s Association, Registered Nurses’ Associations). Finally, key authors on PAB will be contacted to identify non-indexed literature.

### Search strategy

For the database search, initial keywords and descriptors have been determined by the research assistant (MHL) and the principal investigator (AB), with the help of a librarian, for the following three concepts (see Table 1): (1) physically aggressive behaviors, (2) older people, and (3) cognitive impairment. The first round of searching has been done by MHL in CINAHL and PubMed to iteratively refine the keywords and descriptors. Once the search strategy is effective, a search with these refined keywords and descriptors will be carried out in the other databases and websites mentioned above.

**Table 1:** Major concepts and related initial keywords used for building the search strategy

Concept 1	Concept 2	Concept 3
Physically aggressive behaviors	Older people	Cognitive impairment
Abusive behavio* Acting out Aggressi* Aggressive behavio* Aggressive conduct* Aggressive action* Aggressive manner* Aggressive interaction* Agitat* Anger Assault* Assertiv* Attack* Behavioral and psychological symptoms of dementia Behavioral disturbance Behavioral symptom Bit* BPSD Care-resistant Challenging behavior Disruptive behavior Grab/Grabbing Hit* Hostil* Hurt* Impuls* Intrusive behavior Irritab* Kick* Negative behavio* Pok* Provocative behavio* Push* Rag* Reactive behavio* Resistance to care Resistant behavio* Resistiveness to care Responsive behavio* Restlessness Scratch* Spit* Threat* Throw* Uncooperative behavio* Violen*	Aged Elder* adult Elder* people Elder* person Geriatric* Gerontolog* Older adult* Older people Older person* Senior*	Alzheimer* Cognit* decline Cognit* disorder Cognit* dysfunction Cognit*impairment Dementia Frontotemporal Lewy Body Neurocognit* decline Neurocognit*disorder Neurocognit*dysfunction Neurocognit*impairment Vascular dementia Wernicke-Korsakoff Syndrome

Note: Truncation, represented by an asterisk (*), is used to replace missing letters

## Study records

### Data management

The literature from the different data sources will be imported into Endnote^TM^ X9, duplicates will be removed, and the literature will be organized in groups and subgroups. This software will also be used to proceed with the selection process by two independent persons.

### Selection process

First, the literature will be screened with the title and abstract based on the eligibility criteria and the review questions. The literature will be organized as relevant, non-relevant, or of uncertain relevancy. The publications deemed relevant at this stage will be read in full to validate their eligibility. The reasons for exclusions will be documented. This process will be carried out by two independent persons (MHL and AB) for about 20% of the literature identified in the first round of the search to calibrate the selection process. Subsequently, the process will be completed by one person (MHL). The literature tagged as having an uncertain relevancy will be discussed with the research team to reach a consensus. Our clinicians and family caregiver/advocate consultants for this scoping review will be invited to propose additional literature that might be missing from the selected literature. Once the selection process is completed, the same process of the independent review will be carried out for data extraction. A unique identifier will be assigned to each publication during the data extraction.

### Data extraction

Using tables built in Excel^TM^ software, the following data will be extracted from the selected literature:

1. General data: title, year of publication, authors name, discipline and country, type of literature (e.g., primary study, literature review), and aim/research questions of the study;
2. Theoretical data: framework of the publication, if mentioned;
3. Methodological data (if applicable): research design, setting, sampling (size, inclusion/exclusion criteria), participant characteristics (e.g., age, sex, diagnosis), data collection and analysis methods, and limits/strengths as mentioned by the authors;
4. Results data: results on factors, causes, consequences, or interventions associated with the PAB of older people with cognitive impairment, whether these results consider the relationship between the actors and, if so, how.

Although it is not expected of a scoping review [12], we will assess the quality of the literature during the data extraction and will present the results. Four assessment tools will be used: the Mixed Methods Appraisal Tool (MMAT) for primary studies (qualitative, quantitative, mixed) [13], the Revised Assessment of Multiple Systematic Reviews (R-AMSTAR) for systematic reviews [14], the Appraisal of Guidelines for Research and Evaluation II (AGREE II) for clinical practice guides [15], and the Narrative, Opinion, and Text Assessment and Review Instrument (NOTARI) for theoretical or opinion articles [16].

### Data synthesis

Extracted data will be processed using content analysis techniques inspired by Miles, Huberman, and Saldaña [17]. This data analysis method has three steps: (1) data condensation, (2) data display of similarities and differences, and (3) drawing and verifying conclusions (noting themes and subthemes). The results will be presented in narrative form with tables and graphs. Once a first version of the results and recommendations is available, our clinicians and family caregiver/advocate consultants will be invited to enrich the results based on their experience. The final results will be presented in adherence to the Preferred Reporting Items for Systematic Review and Meta-Analysis (PRISMA) [18] adapted to a scoping review.

## Discussion

This project is a direct result of the concerns expressed by formal caregivers and family caregivers during the previous studies conducted by the principal investigator (AB) [19, 20]. These caregivers said they felt helpless in dealing with PAB and that they lacked knowledge about these behaviors. They did not understand the causes of these behaviors or the most effective and human way to prevent or manage them. So far, no systematic review seems to have determined the state of knowledge of the causes, associated factors, consequences, and interventions for this type of behavior. By including a humanist framework specific to people with cognitive impairment, this scoping review will identify research areas that call for further development so that the particular needs of this vulnerable population may be taken into account. As the data extraction will include an assessment of the quality of the literature, it will be possible to discuss the quality of existing knowledge in the development of a research agenda on PAB. This addition to the method is in response to the criticism that is sometimes formulated of scoping reviews and that may reduce the usefulness of their results [6, 21, 22]. By adding this dimension to our data extraction, it will also be possible to discuss the relevance of the current state of knowledge. Furthermore, the results of this scoping review will ascertain what knowledge could be transferred to clinical practice during the initial training and continuing education of healthcare professionals. Depending on the results, recommendations for the organization of home, hospital, and residential care and services could be made. In conclusion, the results of this scoping review will guide the development of a research agenda, of clinical knowledge, and of the training on the best practices to assess, prevent, and intervene when older persons with cognitive impairment manifest PAB.

## Additional file

Additional file 1: Preferred reporting items for systematic review and meta-analysis protocols (PRISMA-P). Checklist items for systematic review protocols (administrative information, introduction and methods). (DOCX 33 kb)

## Data Availability

Not applicable.

## Abbreviations

AGREE II: Appraisal of Guidelines for Research and Evaluation II
CINAHL: Cumulative Index to Nursing and Allied Health Literature
MMAT: Mixed Methods Appraisal Tool
NOTARI: Narrative, Opinion and Text Assessment and Review Instrument
PAB: Physically aggressive behaviors
PRISMA: Preferred Reporting Items for Systematic Review and Meta-Analysis
PRISMA-P: Preferred Reporting Items for Systematic Review and Meta-Analysis Protocols
PROSPERO: International Prospective Register of Systematic Review
R-AMSTAR: Revised Assessment of Multiple Systematic Reviews
